# Biocatalytic Enantioselective Synthesis of Atropisomers

**DOI:** 10.1021/acs.accounts.2c00572

**Published:** 2022-11-07

**Authors:** Olivia F. B. Watts, Jordan Berreur, Beatrice S. L. Collins, Jonathan Clayden

**Affiliations:** School of Chemistry, University of Bristol, Bristol BS8 1TS, U.K.

## Abstract

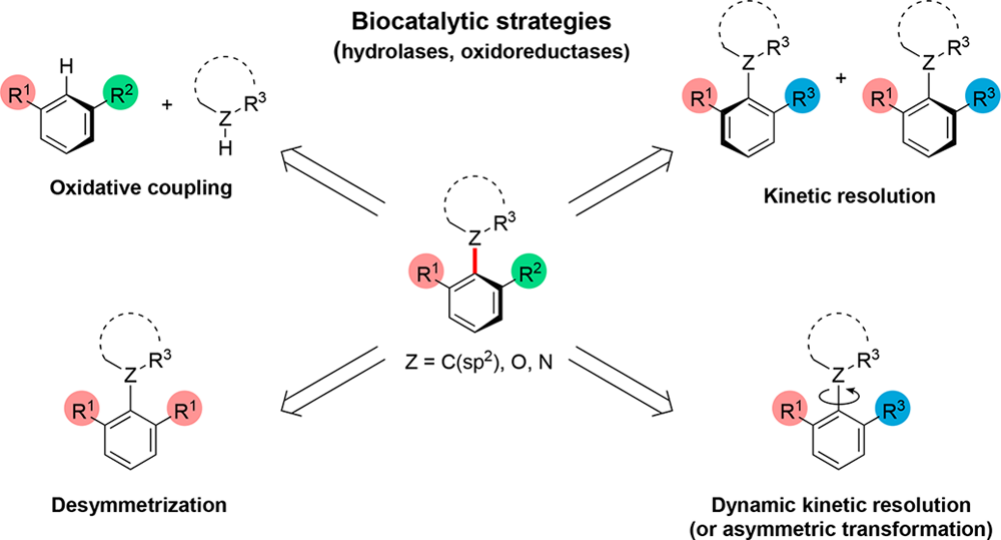

Atropisomeric compounds are found extensively as natural products, as ligands for asymmetric transition-metal catalysis, and increasingly as bioactive and pharmaceutically relevant targets. Their enantioselective synthesis is therefore an important ongoing research target. While a vast majority of known atropisomeric structures are (hetero)biaryls, which display hindered rotation around a C–C single bond, our group’s long-standing interest in the control of molecular conformation has led to the identification and stereoselective preparation of a variety of other classes of “nonbiaryl” atropisomeric compounds displaying restricted rotation around C–C, C–N, C–O, and C–S single bonds.

Biocatalytic transformations are finding increasing application in both academic and industrial contexts as a result of a significant broadening of the range of biocatalytic reactions and sources of enzymes available to the synthetic chemist. In this Account, we summarize the main biocatalytic strategies currently available for the asymmetric synthesis of biaryl, heterobiaryl, and nonbiaryl atropisomers. As is the case with more traditional synthetic approaches to these compounds, most biocatalytic methodologies for the construction of enantioenriched atropisomers follow one of two distinct strategies. The first of these is the direct asymmetric construction of atropisomeric bonds. Synthetically applicable biocatalytic methodologies for this type of transformation are limited, despite the extensive research into the biosynthesis of (hetero)biaryls by oxidative homocoupling or cross-coupling of electron-rich arenes. The second of these is the asymmetric transformation of a molecule in which the bond that will form the axis already exists, and this approach represents the majority of biocatalytic strategies available to the synthetic organic chemist. This strategy encompasses a variety of stereoselective techniques including kinetic resolution (KR), desymmetrization, dynamic kinetic resolution (DKR), and dynamic kinetic asymmetric transformation (DYKAT).

Nondynamic kinetic resolution (KR) of conformationally stable biaryl derivatives has provided the earliest and most numerous examples of synthetically useful methodologies for the enantioselective preparation of atropisomeric compounds. Lipases (i.e., enzymes that mediate the formation or hydrolysis of esters) are particularly effective and have attracted broad attention. This success has led researchers to broaden the scope of lipase-mediated transformations to desymmetrization reactions, in addition to a limited number of DKR and DYKAT examples. By contrast, our group has used redox enzymes, including an engineered galactose oxidase (GOase) and commercially available ketoreductases (KREDs), to desymmetrize prochiral atropisomeric diaryl ether and biaryl derivatives. Building on this experience and our long-standing interest in dynamic conformational processes, we later harnessed intramolecular noncovalent interactions to facilitate bond rotation at ambient temperatures, which allowed the development of the efficient DKR of heterobiaryl aldehydes using KREDs. With this Account we provide an overview of the current and prospective biocatalytic strategies available to the synthetic organic chemist for the enantioselective preparation of atropisomeric molecules.

## Key References

- YuanB.; PageA.; WorrallC. P.; EscalettesF.; WilliesS. C.; McDouallJ. J. W.; TurnerN. J.; ClaydenJ.Biocatalytic Desymmetrization of an Atropisomer with Both an Enantioselective Oxidase and Ketoreductases. Angew. Chem., Int. Ed.2010, 49( (39), ), 7010–701310.1002/anie.20100258020715245.^1^*Enantiomerically enriched atropisomeric diaryl ethers are synthesized by desymmetrization, either by the enzymatic atroposelective oxidation of a benzylic hydroxy group or by the enzymatic atroposelective reduction of an aldehyde.*
- StanilandS.; AdamsR. W.; McDouallJ. J. W.; MaffucciI.; ContiniA.; GraingerD. M.; TurnerN. J.; ClaydenJ.Biocatalytic Dynamic Kinetic Resolution for the Synthesis of Atropisomeric Biaryl N-Oxide Lewis Base Catalysts. Angew. Chem., Int. Ed.2016, 55( (36), ), 10755–1075910.1002/anie.20160548627504722.^2^*Dynamic kinetic resolution of rapidly racemizing biarylpyridine and isoquinoline N-oxide derivatives affords enantiomerically enriched conformationally stable products via KRED-mediated stereoselective reduction.*

## 1 Introduction

**Figure 1:**
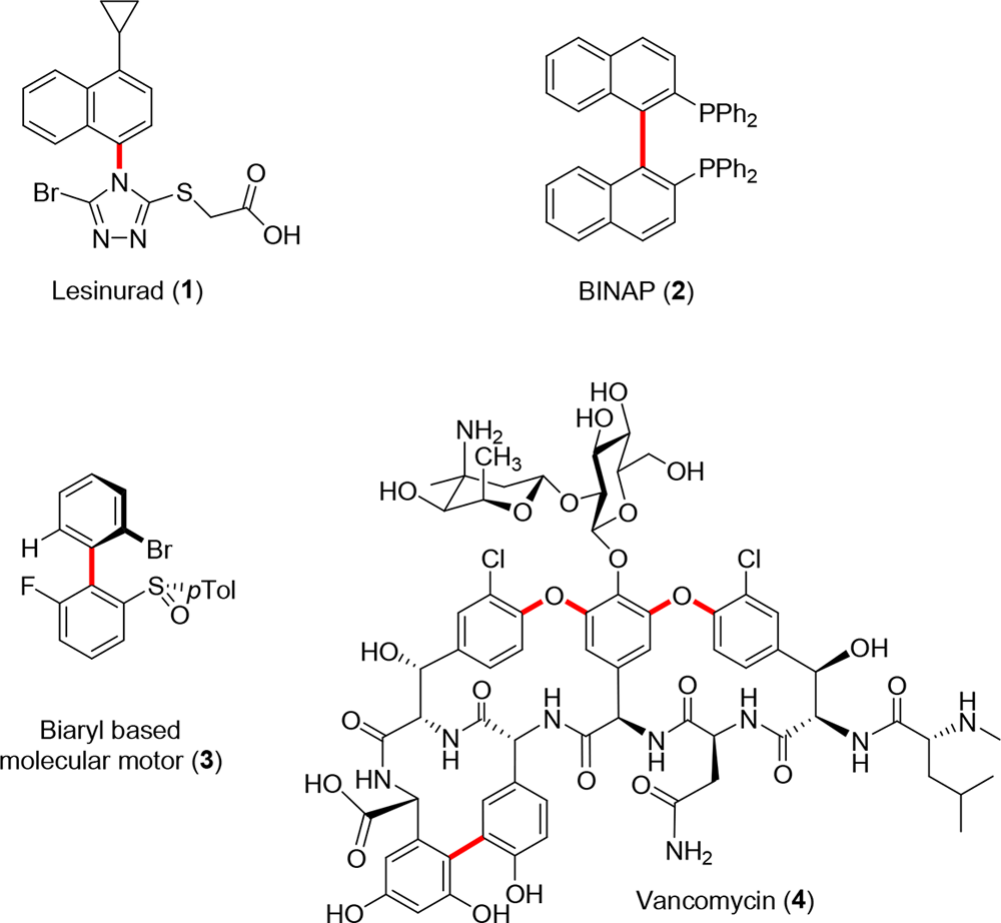
Examples of important atropisomeric molecules. Atropisomeric bonds are highlighted in red.

Atropisomerism is a temperature-dependent molecular property that arises from restricted rotation around a single bond, leading to the emergence of separable conformers. As a consequence of this phenomenon, chirality arises in certain classes of molecules even in the absence of a stereogenic center. Atropisomeric moieties frequently constitute crucial structural elements in bioactive compounds,^3,4^ chiral ligands and organocatalysts,^5,6^ and molecular machines^7^ as well as many natural products such as the antibiotic vancomycin (Figure 1).^8^ This has prompted efforts to develop methods for the enantioselective synthesis of atropisomeric compounds,^9^ including work from our own group on the synthesis and conformational analysis of atropisomers. Our work has explored both (hetero)biaryl compounds displaying hindered rotation around a C–C single bond, as well as rarer types of non-biaryl atropisomeric compounds such as benzamides (C–C atropisomers),^10^ anilides, urea and diarylamine derivatives (C–N atropisomers),^11−14^ diaryl ethers (C–O),^15^ and diaryl sulfones (C–S).^16,17^

The most common, but also most challenging, synthetic strategy for the preparation of enantioenriched atropisomeric compounds is the direct asymmetric construction of the stereogenic axis by aromatic ring formation or the coupling of unsymmetrical partners (homo- or heterocoupling). However, several strategies for atropisomer synthesis exist in which asymmetric induction occurs after the formation of the atropisomeric bond. These include kinetic resolution (KR), desymmetrization, dynamic kinetic resolution (DKR), and dynamic kinetic asymmetric transformation (DYKAT).^9,18,19^ In addition, our group has reported the development of various methods for synthesizing atropisomeric compounds using dynamic thermodynamic resolution^10,20^ assisted by various chiral auxiliaries as well as the desymmetrization of prochiral molecules.

More recently, we have developed the use of enzymes in the atroposelective synthesis of biaryl compounds, exploiting the high enantioselectivities, mild conditions, and high turnover rates associated with biological catalysts.^21,22^ In this Account, we will review the use of biocatalysis in the synthesis of enantioenriched atropisomeric compounds. Despite the wide variety of enzymatic transformations employed in primary and secondary metabolism and especially in the biosynthesis of natural products, biological catalysts have been applied only to a limited repertoire of transformations in synthetic organic chemistry. Among these, the subset of enzymes that have been employed in the synthesis of atropisomeric compounds is even more restricted. We shall describe reported biocatalytic methods for the synthesis of atropisomers, including the use of oxidoreductase and hydrolase enzymes across a range of synthetic strategies comprising oxidative coupling, KR, desymmetrization, DKR, and DYKAT. We conclude by discussing future prospects for the application of biocatalysts to the enantioselective synthesis of atropisomers.

## 2 Oxidative Coupling

The direct asymmetric coupling of two unsymmetrical partners seems to be an appealing strategy when devising the synthesis of an enantioenriched atropisomeric product. However, such reactions are often subject to high kinetic barriers, especially in the synthesis of sterically demanding tetra-*ortho*-substituted biaryls. Such direct coupling reactions require the use of high temperatures, often at the expense of already nontrivial control of the stereochemical outcome.^9,19^ Additionally, most cross-coupling methods require appropriate functionalization of at least one of the coupling partners to ensure control of the regioselectivity.

The synthetically appealing coupling of two unfunctionalized partners (i.e., the coupling of two C–H bonds to form a new C–C bond) is a strategy commonly employed by nature for the construction of atropisomeric natural products.^23,24^ Existing nonenzymatic methodologies for such direct oxidative couplings are limited by substrate selectivity (dimerization vs cross-coupling) as well as chemoselectivity, stereoselectivity, and regioselectivity (i.e., the ability to distinguish between a number of C–H bonds of similar reactivity).^25^ Enzymes, on the other hand, benefit from the complex 3D structure of their active site to ensure efficient templating of the two substrates to achieve high levels of selectivity.^26^

Many enzymes, including laccases, peroxidases, or cytochrome P450 monooxygenases, catalyze the oxidative coupling of electron-rich arenes (usually phenol derivatives) to deliver atropisomeric products with high chemo- and regioselectivity as well as stereoselectivity.^24^ Interestingly, the intervention of additional enzymes and/or so-called dirigent proteins^27^ (i.e., proteins that are catalytically inactive but exert control over the regioselectivity and stereochemical outcome of bimolecular oxidative couplings) is sometimes required to achieve the high selectivities observed in the atroposelective biosynthesis of some secondary metabolites. While many biosyntheses of atropisomerically enriched natural products by oxidative coupling have been uncovered, few examples of the application of the enzymes responsible for this crucial step in broader synthetic chemistry have been reported to date.^26^

**Scheme 1:**
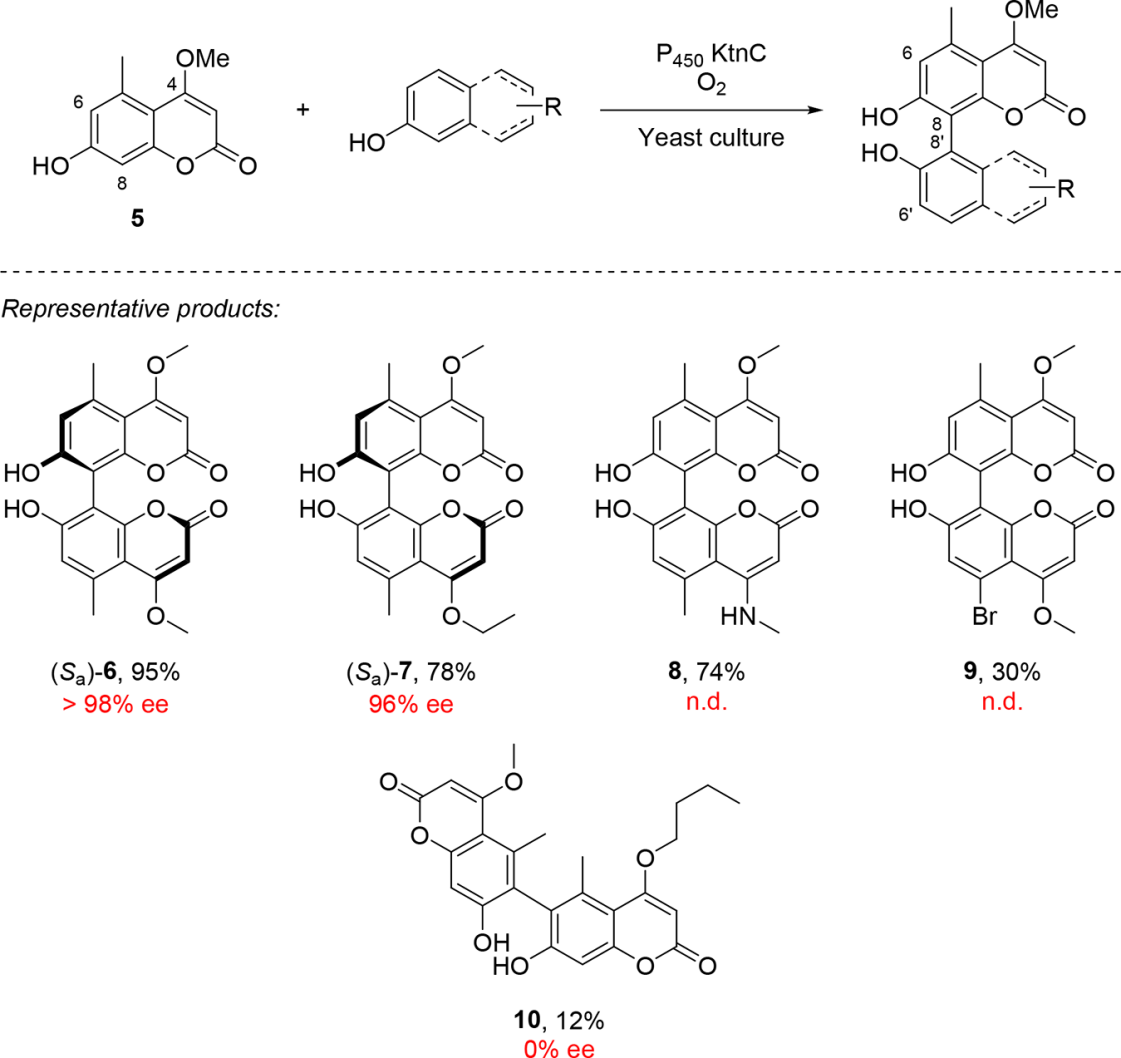
Cytochrome P450 Enzyme-Mediated Atroposelective Oxidative Homocoupling and Cross-Coupling of Its Native Substrate **5** with Phenol Derivatives

Narayan and co-workers reported the use of cytochrome P450 enzyme KtnC expressed in *Pichia pastoris* for the homocoupling of native substrate **5** and its cross-coupling with a range of phenol derivatives to achieve non-natural biaryl products **6**–**10** (Scheme 1).^28^ While the substrate scope is relatively extensive, the stereochemical outcome was reported only in a handful of cases. Remarkably, KtnC shows consistent regioselectivity in these couplings, achieving only the 8,8′-connected products. In a few cases, however, the 6,6′-connected product is obtained (even preferentially in the case of **10**), albeit with lower levels of stereocontrol. In addition to this work with the wild-type P450 enzyme, the authors developed a strategy for its directed evolution, leading to greatly improved conversion and regioselectivity on an originally poorly responsive substrate. Further rounds of engineering led to increased stereoselectivity for the same transformation, though at the expense of yield.

## 3 Kinetic Resolution

**Scheme 2:**
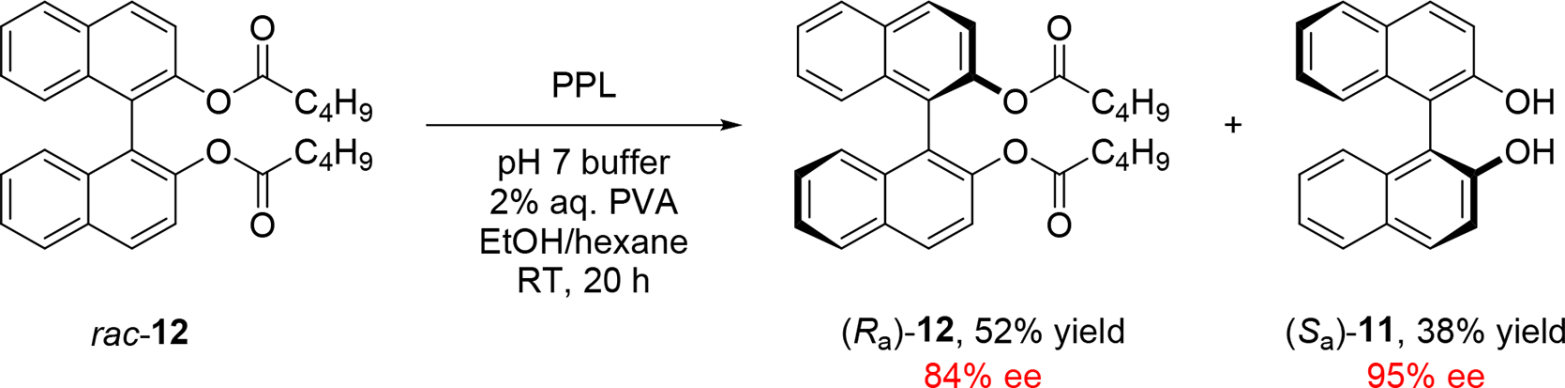
Early Example of Biocatalytic Atroposelective Ester Hydrolysis Reported by Miyano and Co-workers, in Which Valeric Diester *rac*-**12** Was Selectively Hydrolyzed to Afford (*S*_a_)-BINOL **11**

A majority of enzymatic atroposelective syntheses reported have employed lipases, commensurate with their routine use in organic synthesis. Their widespread use owes its origin to their wide substrate range, high tolerance of organic solvents, and the enantioselectivity they display during acylation, deacylation, and acyl transfer reactions under mild conditions.^29^ An early example of biocatalytic KR of atropisomers was reported by Ikekawa and co-workers in 1985, who obtained samples of 2,2′-dihydroxy-1,1′-binaphthyl (BINOL) (**11**) in enantiomeric excess of up to 96% by the deacylation of various diester derivatives using microorganism extracts.^30^ Miyano and co-workers later showed that commercially available lipases such as porcine pancreatic lipase (PPL) were also suitable for this purpose (Scheme 2),^31^ leading to the routine use of lipases to atroposelectively esterify dihydroxy-binaphthyl and dicarboxylic acid binaphthyl derivatives^32−37^ as well as to hydrolyze aza-BINOL derivatives of binaphthyl compounds.^38,39^

**Scheme 3:**
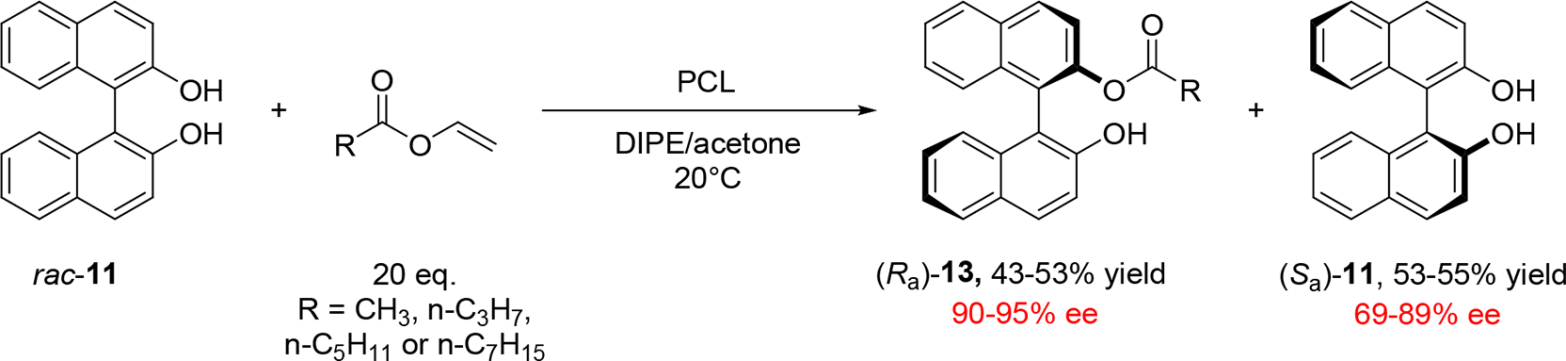
Early Example of Biocatalytic KR of *rac*-BINOL **11***via* Acylation by a Variety of Vinyl Ester Acyl Donors to Form Monoesters **13**

BINOL-based substrates were also among the first to be subjected to atroposelective lipase-catalyzed acylation. In 1989, Oda and co-workers reported the use of the atroposelective lipase-catalyzed acylation of BINOL and the deacylation of its esters in a solely organic solvent system (Scheme 3), catalyzed by *Pseudomonas cepacia* lipase (PCL). This demonstrated the ability of lipases to catalyze the transesterification of alcohols from a suitable acyl donor in the absence of water, the presence of which generally also induces competitive hydrolysis to occur.^40^

**Scheme 4:**
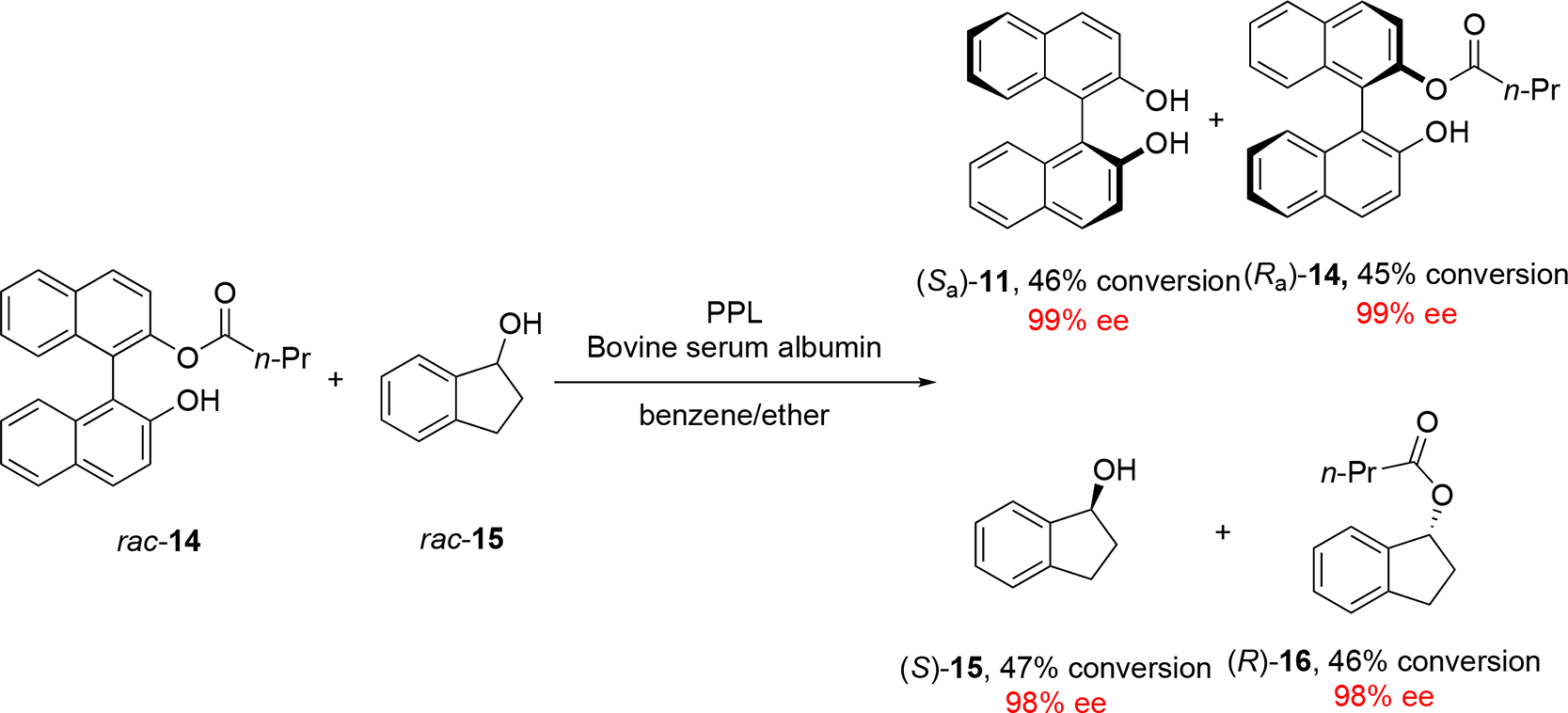
PPL-Catalyzed Stereoselective Transesterification of Butyl Ester **14** and Indanol **15**

Oda’s acylation employed a range of vinyl esters whose use as acyl donors in lipase-catalyzed transesterification reactions arises from their ability to push reaction equilibria toward the desired ester formation, as the enol byproducts irreversibly tautomerize to the corresponding aldehyde. A more recent example of lipase-mediated atroposelective acylation reported by Akai and co-workers was accelerated by the addition of Na_2_CO_3_.^41^ Acyl transfer is not restricted to vinyl esters, as a wide variety of compounds can behave as acyl donors. Sun and co-workers showed that biaryl esters themselves can also act as acyl donors during a lipase-catalyzed transesterification between biaryl-derived ester **14** and indanol **15** in the presence of PPL enzyme, affording esterified indanol derivative **16** in 99% ee and 45% conversion (Scheme 4).^42^

**Scheme 5:**
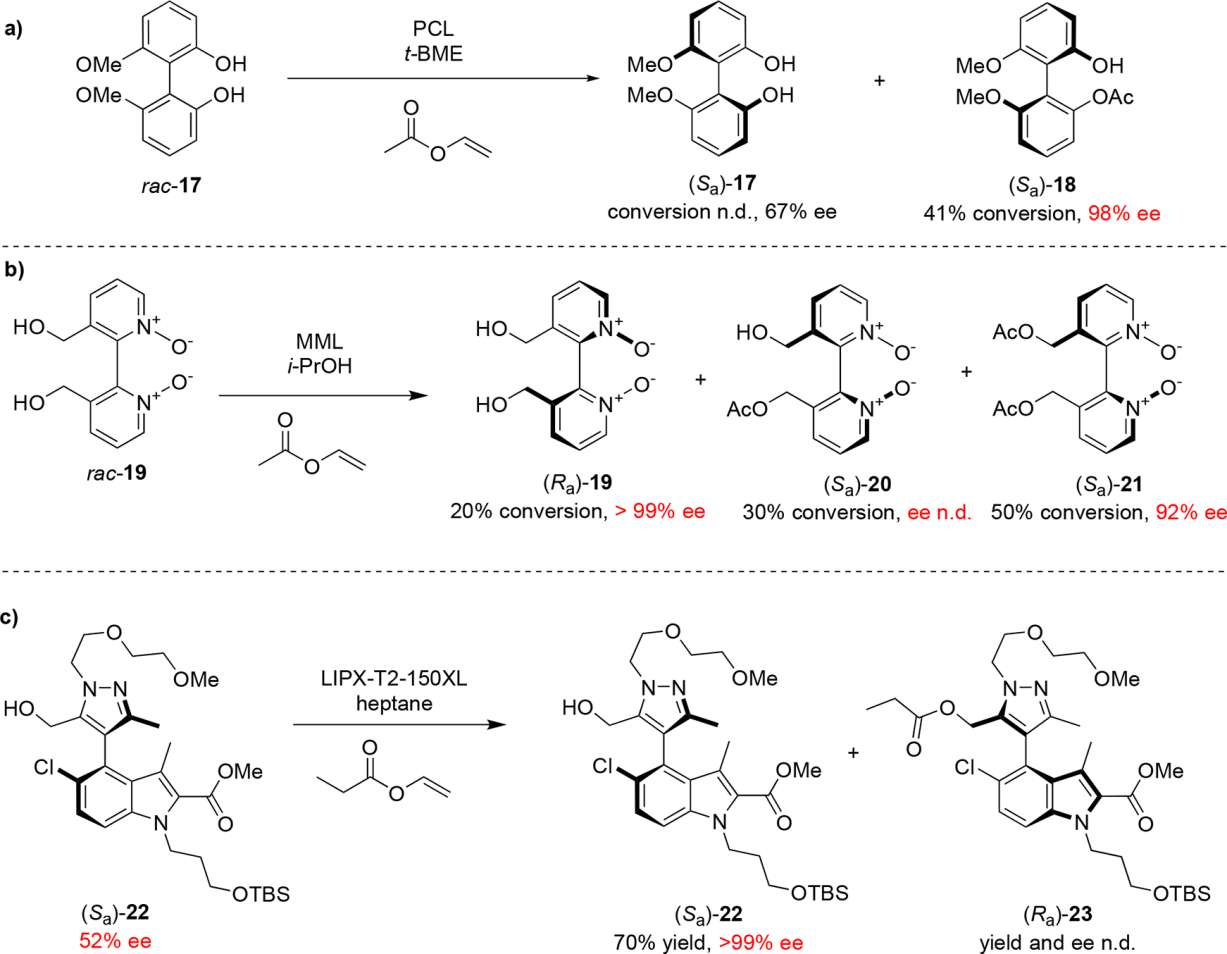
(a) Early Example of a Biocatalytic KR of a Biphenyl Diol *rac*-**17** by PCL-Mediated Acylation, (b) KR of Bipyridinyl *N*-Oxide Atropisomers by MML-Mediated Acylation, and (c) Further Enantioenrichment of Synthetic Intermediate (*S*_a_)-**22** during the Asymmetric Synthesis of JNJ-4355 by Biocatalytic Acylation

The scope of atroposelective biocatalytic KR was later widened to encompass biphenyls, with Sanfilippo and co-workers reporting highly enantioselective enzymatic acylations of dihydroxylated biphenyl **17** and bipyridinyl *N*-oxide substrate **19** in the presence of PCL and *Mucor miehei* lipase (MML) enzymes (Scheme 5a,b).^43,44^ Several examples of KRs of dihydroxylbiphenyl ester derivatives via lipase-catalyzed hydrolysis have also since been reported, such as the enantioselective hydrolysis of a hexamethylbiphenol ester derivative by PPL^45^ and the enzymatic kinetic resolution of atropisomeric intermediate **22** during the asymmetric synthesis of JNJ-4355, an inhibitor of a protein associated with myeloid leukemia (Scheme 5c).^46^

**Scheme 6:**
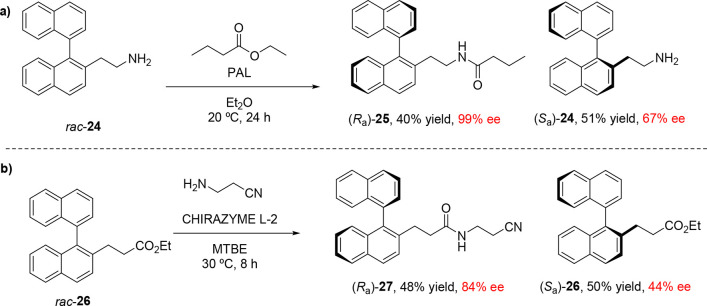
(a) Lipase-Catalyzed Atroposelective Amidation Reactions of Binaphthyl-Based Amines and (b) Biocatalytic Enantioselective Transamidation of Binaphthyl-Based Carboxylate Esters

While most work on the lipase-catalyzed KR of atropisomers has focused on the acylation of alcohols and diacylation of esters, there are limited examples of atroposelective transformations of other functional groups. Examples of biocatalytic amidation are less common than esterification as the higher nucleophilicity of amines compared to alcohols can lead to background nonstereoselective *N*-acylation, necessitating the careful selection of acylating agents for these reactions. An example of atroposelective amidation was reported by Aoyagi and co-workers in 2002 (Scheme 6), in which various 1,1-binaphthylamines were derivatized to the respective amides in the presence of immobilized *Pseudomonas aeruginosa* lipase (PAL).^47^ The reactivity of these substrates to acylation is highly dependent on the length of the alkyl chain between the aromatic ring and the amine reactive site. Out of the substrates investigated, amine **24**, in which the binaphthyl core and amine were separated by an ethylene linker, was most amenable to *N*-acylation to corresponding amide **25** (Scheme 6a). A similar effect was observed in the transamidation of binaphthyl-based esters: phenyl and benzylic carboxylate esters were reluctant substrates for transamidation, but the desired amide **27** could be formed biocatalytically from **26** in 48% yield and 84% ee (Scheme 6b).^48^

**Scheme 7:**
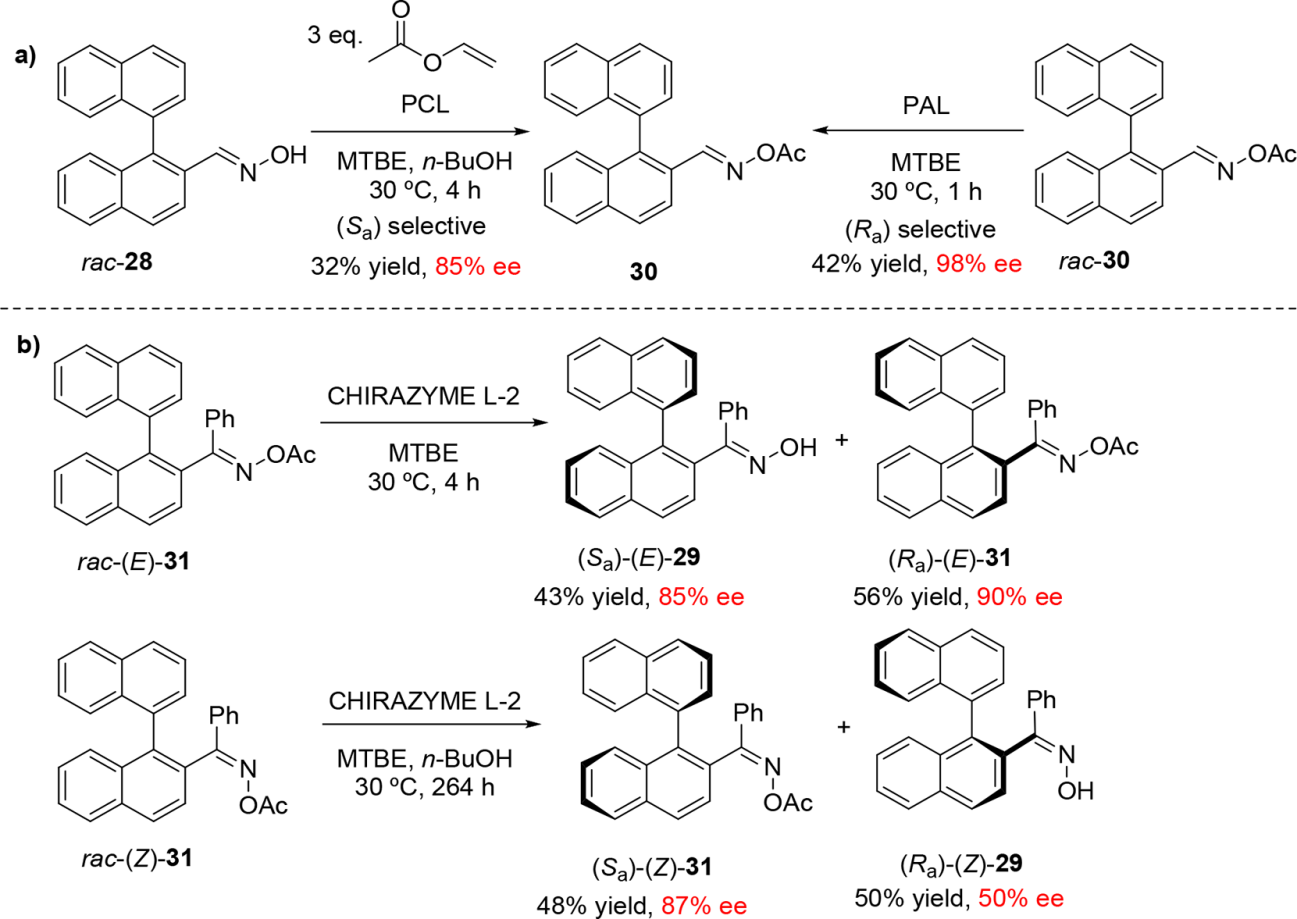
(a) Kinetic Resolutions of Acetylated Binaphthyl Oxime Derivatives by Atroposelective Enzymatic Hydrolysis and (b) Enantiopreference of the CHIRAZYME-L2-Mediated Hydrolysis of **31** Based on the *E* and *Z* Configurations of the Ketoxime Double Bond

Aoyagi and co-workers also investigated the enantioselective lipase-catalyzed acylation and hydrolysis of binaphthyl oxime derivatives **28**, (*E*)-**29**, and (*Z*)-**29** (Scheme 7).^49^ While **28** was amenable to KR by either the lipase-mediated acylation of aldoxime **28** or the deacylation of acetylated derivative **30**, ketoxime **29** was unsuitable for biocatalytic acylation. Interestingly, however, the reactivity of **31** toward lipase-catalyzed hydrolysis was highly dependent on the geometry of the ketoxime: hydrolysis of (*E*)-**31** by CHIRAZYME L-2 afforded the (*S*_a_)-enantiomer of (*E*)-**29** in 85% ee, while the hydrolysis of (*Z*)-**31** afforded (*R*_a_)-(*Z*)-**29** in 50% ee after a much longer reaction time (264 vs 4 h).

**Scheme 8:**
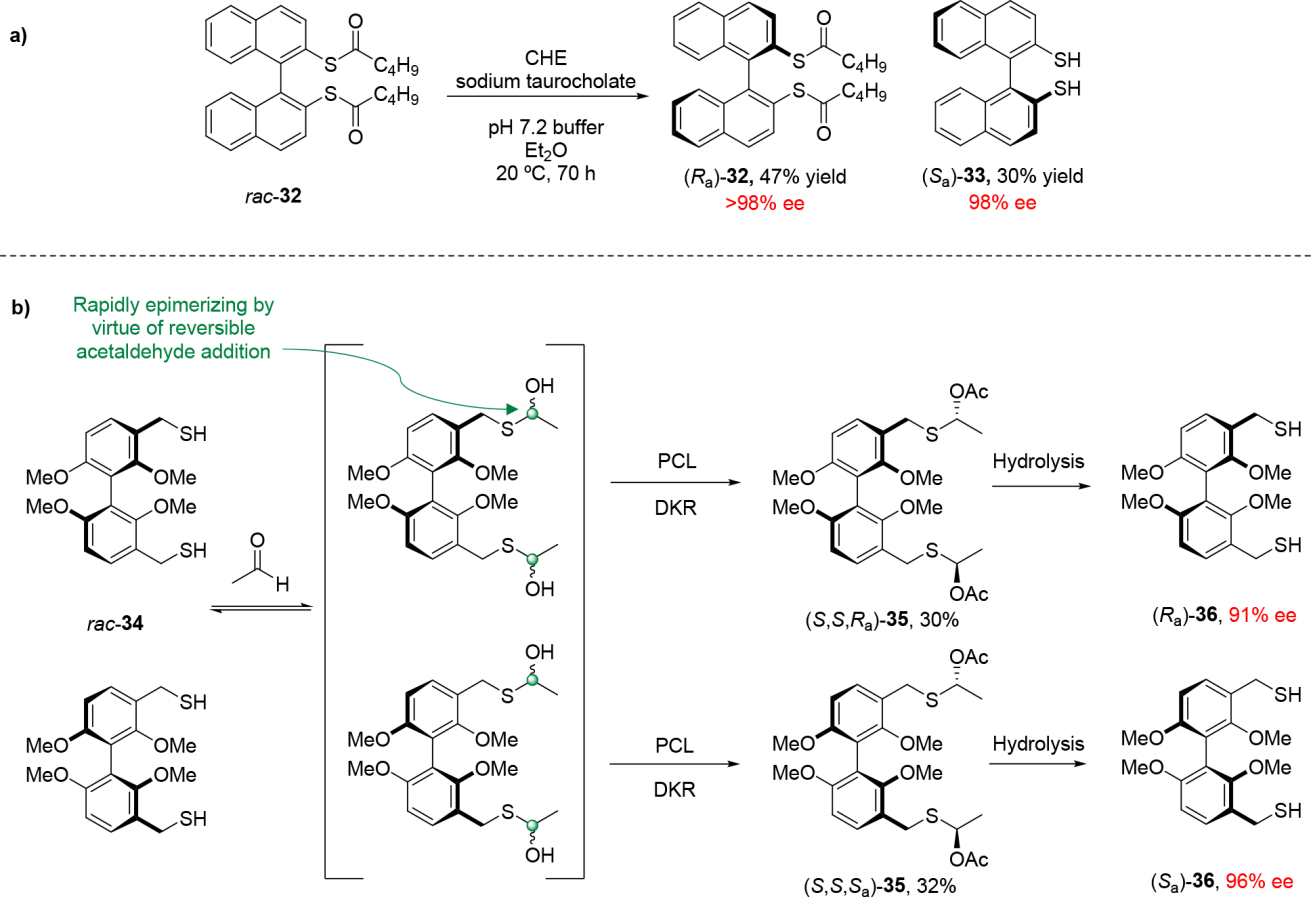
(a) Atroposelective Hydrolytic KR of Thioester **32** in the Presence of Cholesterol Esterase and (b) Enantioseparation of Thiol **34** by Lipase-Catalyzed DKR of Hemithioacetal Groups and Subsequent Hydrolysis

An example of atroposelective thioester hydrolysis was reported by Helmchen and co-workers, in which (1,1′-binaphthalene)-2,2′-dithiol derivative **32** was hydrolyzed to the corresponding thiol **33** in the presence of bovine pancreas acetone powder as a source of cholesterol esterase (CHE) (Scheme 8a).^50^ The enzymatic hydrolysis of biphenyls carrying thioester substituents also occurs in the presence of PCL, but attempts at KR of the equivalent thiol **34** by enzymatic *S*-acylation proved unsuccessful, resulting in thioacetal **35** instead (Scheme 8b).^51^ The lack of reactivity displayed by **34** toward acylation was attributed to the softness of thiol nucleophiles in comparison to alcohols and amines. Observed product **35** was proposed to result from the addition of thiol **34** to the acetaldehyde liberated by the lipase-catalyzed deacylation of vinyl acetate.

**Scheme 9:**
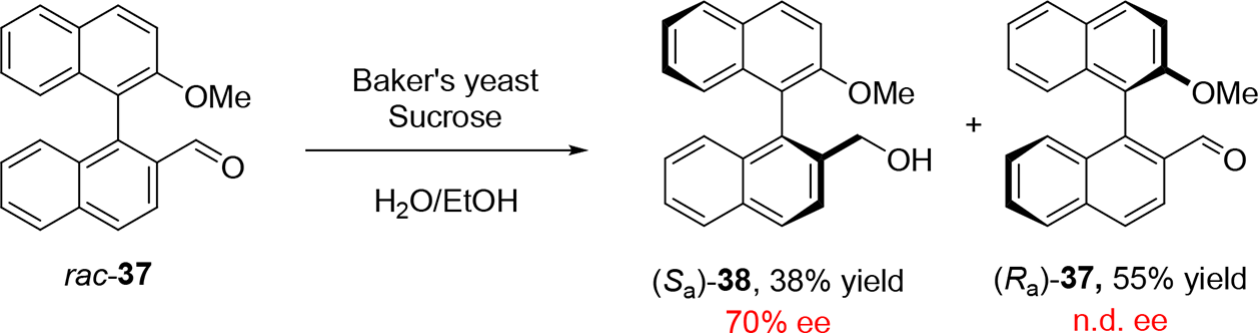
Early Example of the Enantioselective Biocatalytic Reduction of Binaphthyl Aldehydes in the Presence of Baker’s Yeast

Examples of other biocatalytic transformations in KRs of atropisomers remain limited. In an early example of enzymatic atroposelective reduction from Miyano and co-workers in 1988, the reduction of axially chiral binaphthyl-based aldehyde **37** proceeded to the corresponding benzylic alcohol **38** in the presence of baker’s yeast, sucrose, and ethanol (Scheme 9).^52^ In this work, yeast was used as a whole-cell biocatalytic system, exploiting the cell’s native alcohol dehydrogenases (ADHs),^53^ but later examples of biocatalytic atroposelective reductions would be performed with isolated ADHs/ketoreductases (KREDs).^1,2,54^

## 4 Desymmetrization

**Scheme 10:**
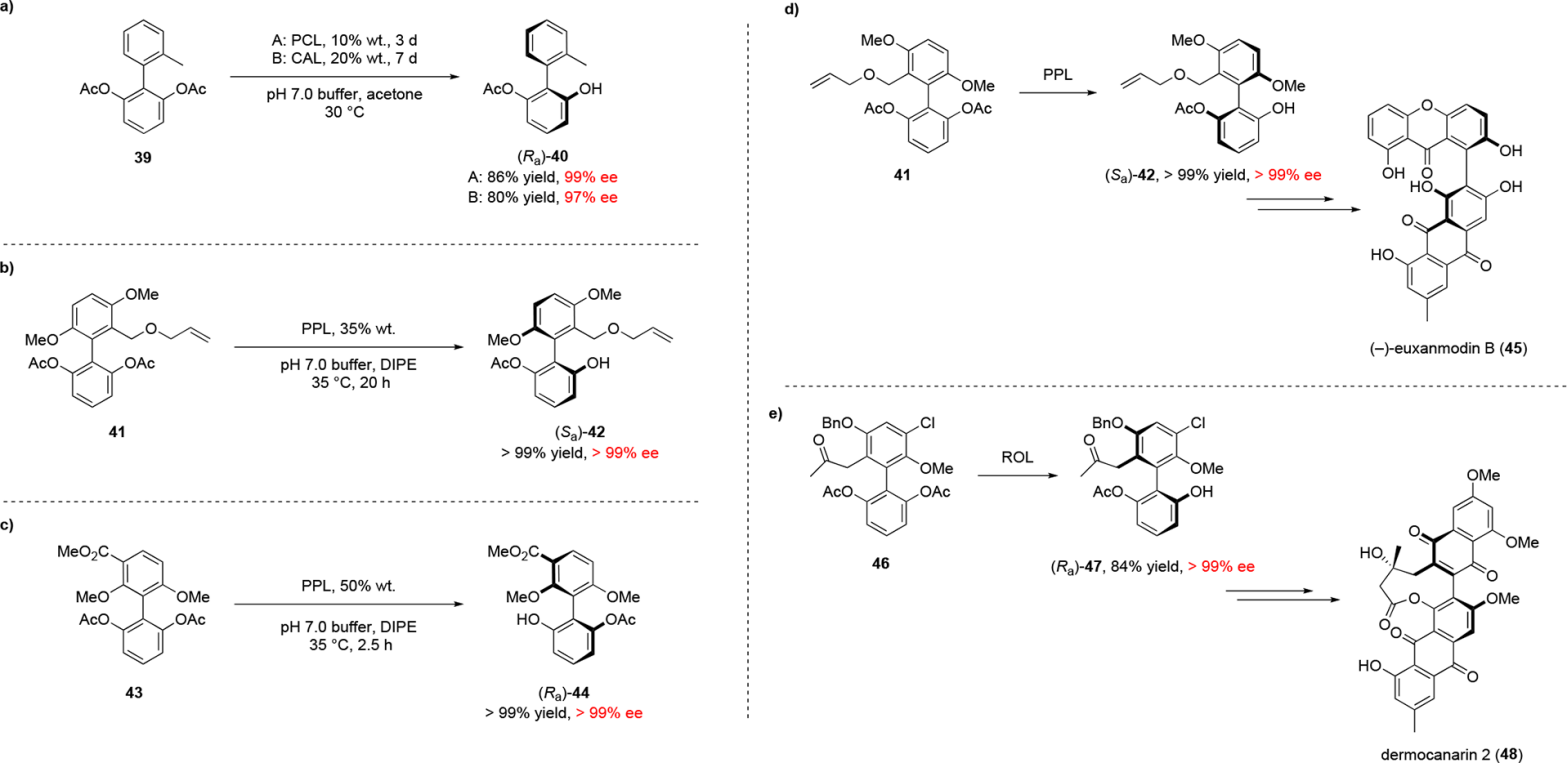
(a–c) Selected Reported Examples of Atroposelective Lipase-Catalyzed Desymmetrization Reactions of Prochiral Biaryl Compounds, Including Highly Hindered Tetra-*ortho*-Substituted Substrates **41** and **43** and (d, e) Examples of Enantioselective Total Syntheses of Atropisomeric Natural Products Employing a Biocatalytic Desymmetrization Step

While KR methods for the synthesis of enantiopure atropisomers have a maximum theoretical yield of 50%, desymmetrization, or the synthesis of a chiral product from a prochiral starting material, allows the synthesis of atropisomers in up to 100% yield and 100% ee. As with KRs, a majority of biocatalytic atroposelective desymmetrizations have been performed using lipase enzymes, with the first example being reported by Matsumoto and co-workers in 2002 as part of an atroposelective desymmetrization of tri-*ortho*-substituted biphenyl diacetates including **39** (Scheme 10a), with the authors later extending the scope of the methodology to include symmetrical tetra-*ortho*-substituted substrates such as **41** (Scheme 10b).^55,56^ The same group also applied this method in the enantioselective total syntheses of (−)-euxanmodin B **45** and dermocanarin 2 **48** to form the desired intermediates in >99% ee using PPL (Scheme 10d,e).^57,58^

The same group later reported that C(3′)-substituents, despite being remote from the biaryl axis, provide sufficient enantiotopic discrimination in biphenyl-2,6-diol diacetate derivatives such as **43** to induce excellent atroposelectivity when subjected to enzymatic hydrolysis (Scheme 10c).^59^ Desymmetrization of similar prochiral biaryl substrates via deacylation and phenol acylation has also been reported by Akai and co-workers, the latter of which was performed using their previously developed base-promoted lipase-catalyzed acylation conditions (Section 3).^60^

**Scheme 11:**
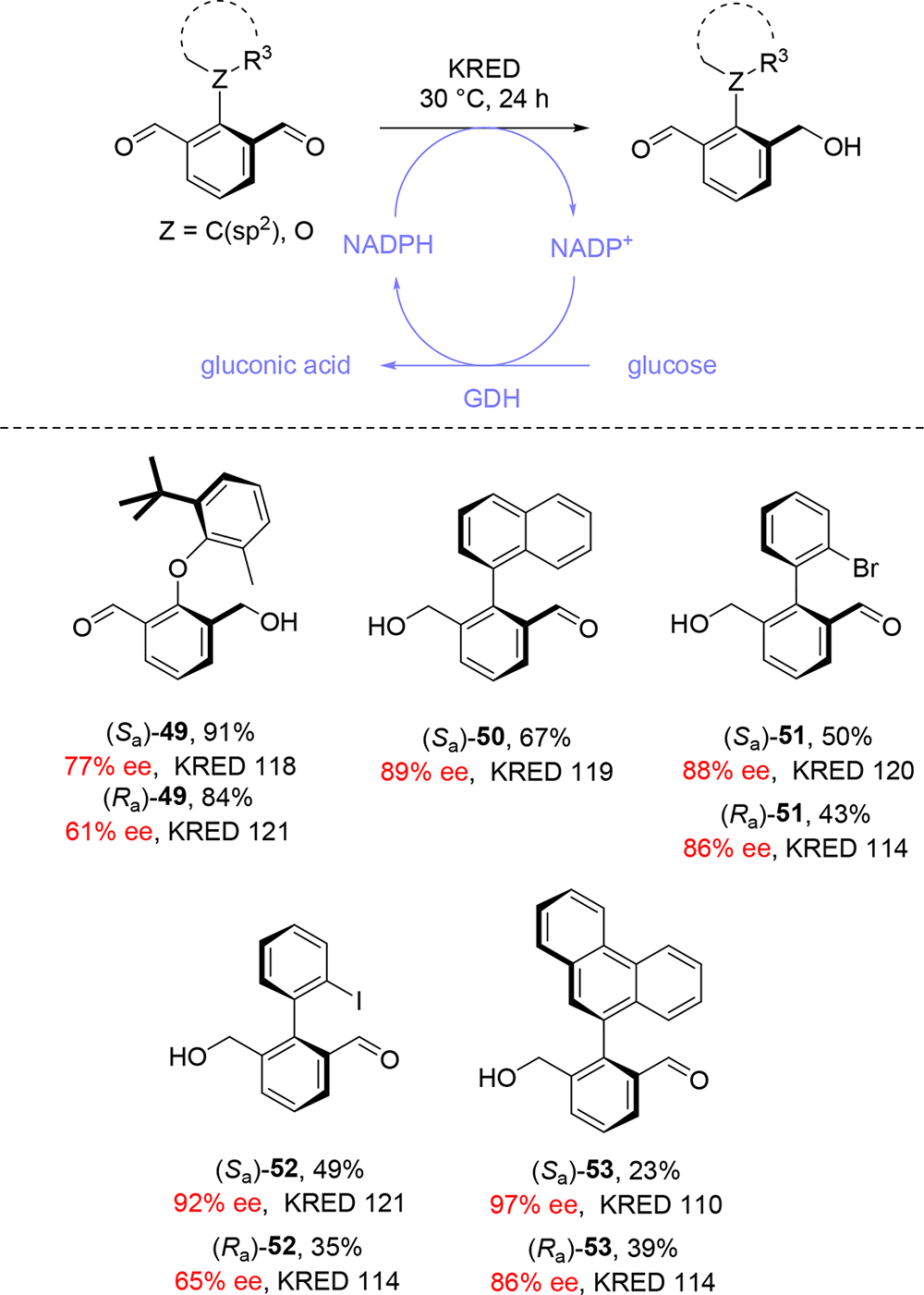
KRED-Mediated Enantioselective Desymmetrization of Pro-atropisomeric Compounds by Aldehyde Reduction

**Scheme 12:**
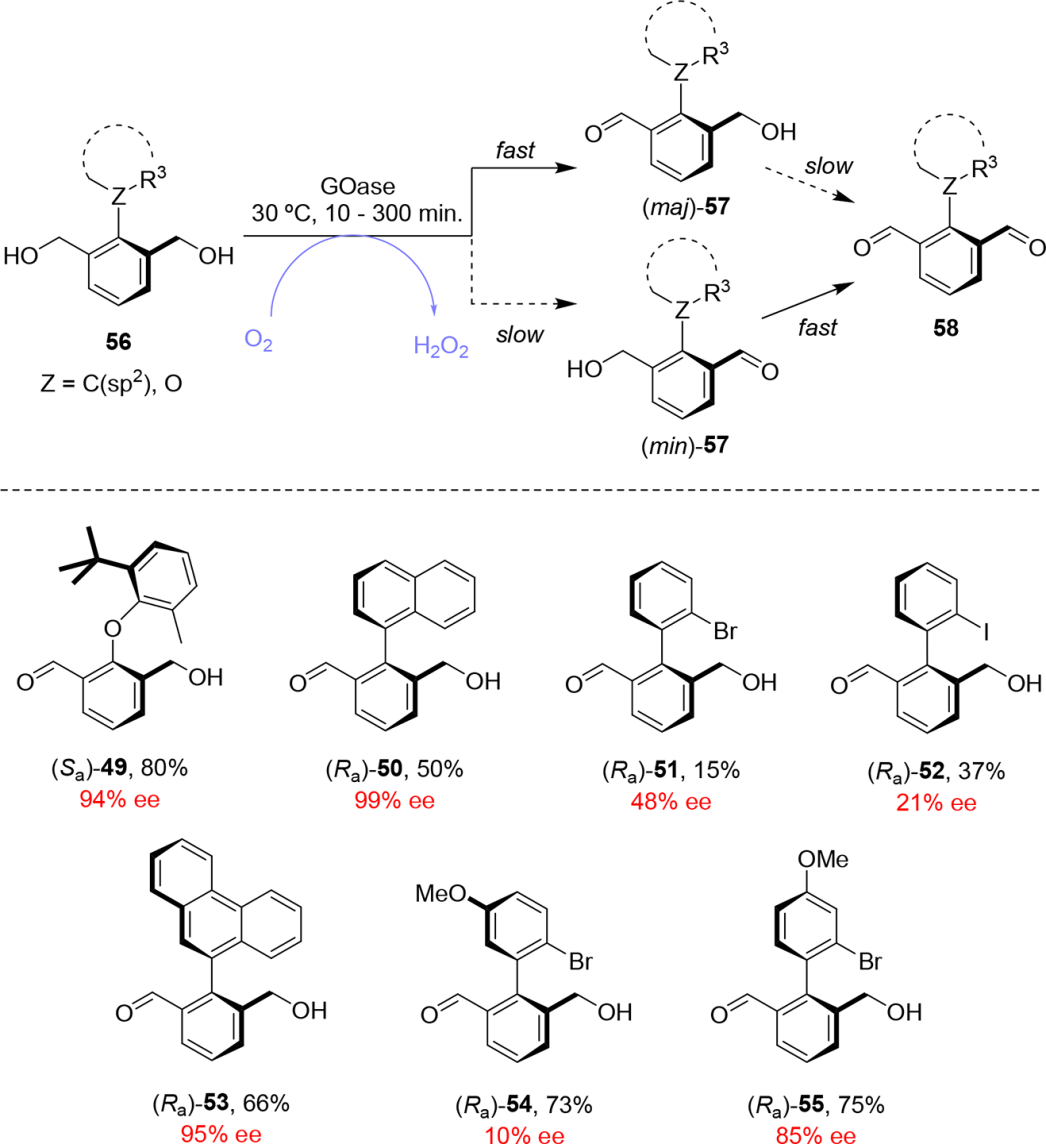
GOase-Mediated Enantioselective Desymmetrization of Pro-atropisomeric Bisbenzylic Alcohol Compounds **56** by Oxidation, Which Can Be Followed by a Second Enantioselective Oxidation to the Corresponding Dialdehyde **58**

Methods for the desymmetrization of pro-atropisomeric compounds mediated by biocatalytic redox enzymes have been developed by members of our group, and we have investigated the use of NAD(P)H-dependent KRED enzymes as enantioselective reducing agents (Scheme 11).^1,54^ In contrast to the work by Miyano and co-workers (Section 3), which employed yeast as a whole cell biocatalyst,^52^ these reductions employed isolated KRED enzymes. This necessitated the incorporation of an NAD(P)H recycling system into the reaction conditions, using glucose as a sacrificial reductant. The activity of KRED enzymes can also be reversed to catalyze alcohol oxidation when this recycling system is adapted to drive the regeneration of NAD(P) rather than NAD(P)H.^61^ Despite the ability of KREDs to function as oxidation catalysts, the oxidative desymmetrization reactions we developed instead employed a mutant galactose oxidase GOase M_3–5_, specifically engineered to accept secondary alcohol substrates (Scheme 12).^62^ This GOase belongs to a different class of oxidases from KREDs, relying on O_2_ as the ultimate sacrificial oxidizing agent.

Both KRED- and GOase-mediated atroposelective desymmetrizations were first reported by members of our group, working with Prof. Nicholas Turner, during the synthesis of atropisomeric diaryl ether derivative **49**.^1^ The diaryl ether linkage is a common motif in natural products such as vancomycin and teicoplanin,^63^ but there were no general asymmetric routes to this class of unusual atropisomer. Subsequent work in the group extended the scope of both the reduction and oxidation methodologies to access a range of monoaldehyde biaryl substrates **50**–**55** in high enantioselectivity (Schemes 11 and 12, respectively). For several substrates, the enantiomer of monoaldehyde obtained depended on the choice of KRED enzyme (Scheme 11).

The GOase-mediated oxidation of pro-atropisomeric compounds carrying two benzylic alcohol groups (generalized as **56** in Scheme 12) also proceeds in high conversion and excellent stereoselectivity. Docking studies revealed insights into the origins of the enantioselectivities of the GOase-mediated oxidations. As is common in desymmetrization reactions, the enantiomeric excesses of the desymmetrization products **57** obtained by GOase oxidation increased over time, as the enantioenrichment of **57** is the result of two stereoselective processes: a desymmetrization and a subsequent KR process. Following enantioselective oxidation of **56** to form monoaldehyde (*maj*)-**57**, further GOase oxidation is also highly stereoselective, resulting in preferential conversion of the minor atropisomer (*min*)-**57** to dialdehyde **58**. This results in further enantioenrichment of **57** over time, albeit at the expense of yield.^64^

## 5 Dynamic Kinetic Resolution and Dynamic Kinetic Asymmetric Transformation

In the earliest example of the use of dynamic kinetic resolution for the asymmetric synthesis of atropisomers, Sanfilippo and co-workers used the DKR of rapidly interconverting hemithioacetal stereocenters to create a pair of atropodiastereomers which were separated chromatographically (Section 3, Scheme 8b).^65^ This work is unique in its use of the DKR of stereocenters remote from the chiral axis to resolve the atropisomers, whereas most atroposelective dynamic resolutions exploit the rotational lability of the (pro)chiral axis. Conformationally labile compounds may undergo transformations that increase their rotational energy barrier sufficiently to lock the axial conformation and create stable atropisomers. Such transformations may be used in DKRs if they proceed stereoselectively and if they are coupled to fast rates of rotation around the prochiral axis in the starting material.

**Scheme 13:**
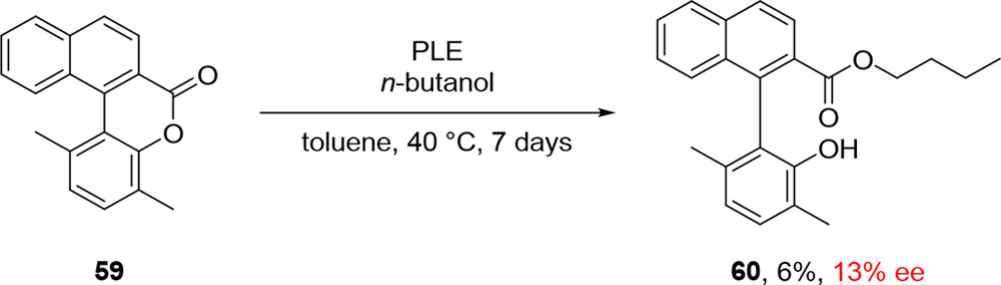
Ring Opening of Lactone **59** Using PLE

Biaryl lactones in which two groups *ortho* to the connecting aryl–aryl bond are linked by an ester bridge usually have a relatively low barrier to Ar–Ar rotation, despite being often tri- or tetra-*ortho*-substituted.^9^ The reduced barrier to rotation is due to the planarization enforced by the bridge which minimizes steric interactions between the *ortho* substituents in the transition state for rotation. The fast interconversion of the enantiomeric lactone conformers allows for the atroposelective cleavage of the lactone bridge by the addition of nucleophiles under dynamic kinetic resolution. Building on the extensive research dedicated to lipase-mediated atroposelective transformation, Deska and co-workers attempted the DKR of lactone **59** by ring opening using various lipases.^66^ This transformation proved sluggish, and only up to 11% conversion to open ester **60** was observed in 0% ee in the presence of *Candida antarctica* type B lipase after 7 days at 40 °C, while a pig liver esterase (PLE) delivered the product with 13% ee and only 6% conversion under the same conditions (Scheme 13).

**Figure 2:**
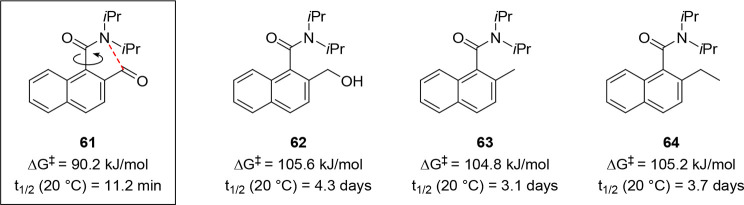
Experimentally determined barriers to rotation and half-lives of racemization at 20 °C of selected 1-naphthamide derivatives. The dashed red line indicates a barrier-lowering n → π* interaction.

Other examples of conformationally labile compounds are 2-formyl-1-benzamide derivatives such as **61** due to the small size and planar nature of their aldehyde substituent as well as an intramolecular noncovalent n → π* interaction between the amide nitrogen lone pair and aldehyde substituent. The barrier to rotation of **61** and derivatives can be dramatically increased by the transformation of the aldehyde into a bulkier, tetrahedral substituent in which the intramolecular interaction no longer exists (Figure 2).^67^ We exploited this property to achieve the dynamic resolution of these compounds under thermodynamic control.^68^

**Scheme 14:**
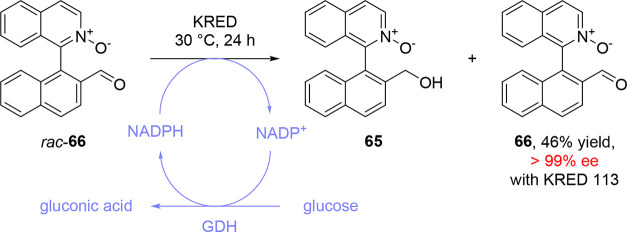
Kinetic Resolution of Biaryl *rac-***66** by KRED-Catalyzed Stereoselective Aldehyde Reduction

Based on this experience, we reasoned that the same type of intramolecular interaction would be present in 2-arylisoquinoline oxide derivative **65**, enabling its preparation by dynamic kinetic resolution. Our investigations started with aldehyde **66**, but this compound quickly proved unsuitable for a dynamic kinetic resolution. In fact, its barrier to rotation was so high that decomposition occurred faster than bond rotation at elevated temperatures. Taking advantage of this conformational stability, **66** was used as a model to determine the ability of commercially available ketoreductases (KREDs) to mediate the stereoselective reduction of the aldehyde function under nondynamic kinetic resolution (Scheme 14).

Absolute configurations of enantioenriched **65** and **66** are unknown.

While the best results were obtained with KRED 113, affording unreacted **66** with 46% yield and >99% ee, other KREDs gave high selectivities, indicating that the enzymes’ active sites were able to accommodate the presence of the atropisomeric biaryl *N*-oxides and distinguish their enantiomers.

**Figure 3:**
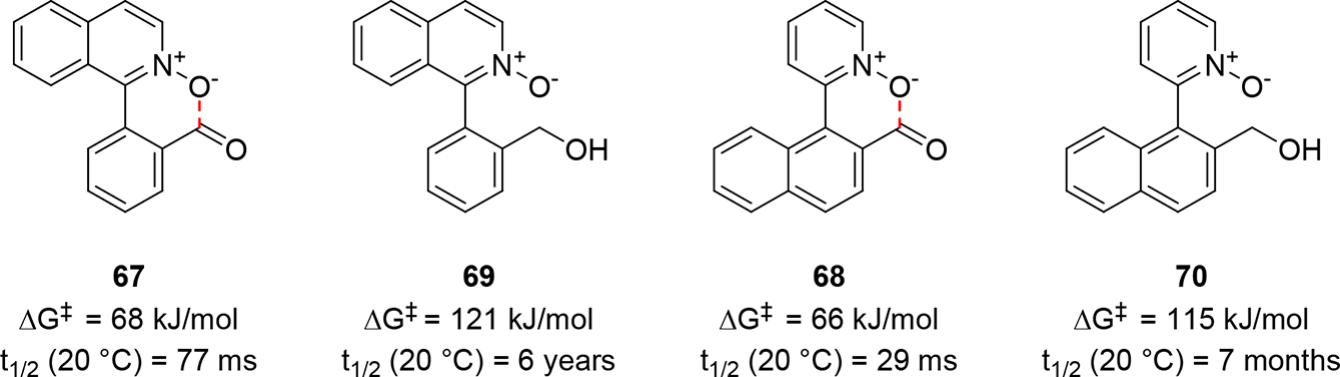
Experimentally determined barriers to rotation and half-lives of racemization at 20 °C of selected biaryl aldehydes and alcohols. The dashed red line indicates a barrier-lowering n → π* interaction.

**Scheme 15:**
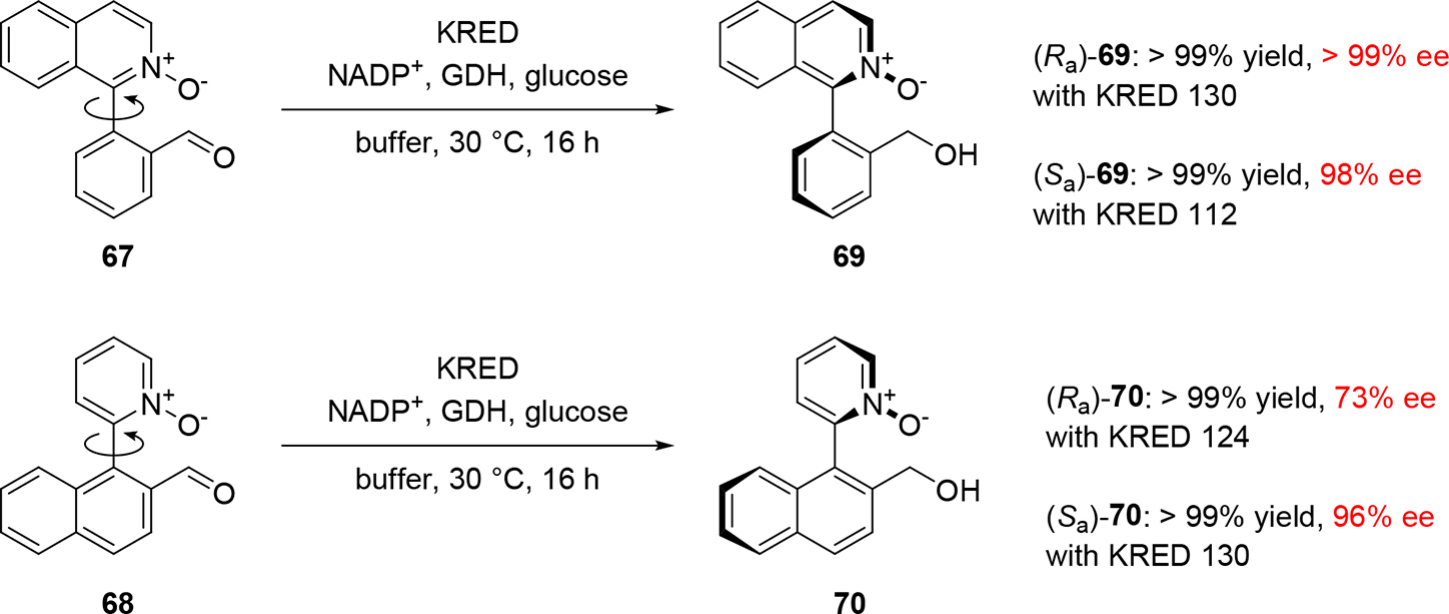
Dynamic Kinetic Resolution of Biaryls **67** and **68** by KRED-Catalyzed Stereoselective Aldehyde Reduction

With the results from this KR in hand, the development of analogous DKR methodology necessitated the study of aldehyde compounds with much lower barriers to enantiomerization. Heterobiaryl *N*-oxides **67** and **68** were prepared, and their rotational barriers were experimentally determined to be 68 and 66 kJ/mol, respectively (i.e., they possess half-lives of racemization shorter than 100 ms at 25 °C). By contrast, their alcohol counterparts **69** and **70** were shown to have much higher barriers to rotation and to be conformationally stable for at least several months at room temperature (Figure 3). When compounds **67** and **68** were screened against a range of KREDs, dynamic kinetic resolution took place with excellent yields and stereoselectivities (Scheme 15).

Catalysis using KRED 130 gave the (*R*_a_) enantiomer of **69** and the (*S*_a_) enantiomer of **70** quantitatively and with excellent stereocontrol. The other enantiomers of both **69** and **70** could be obtained with similarly high efficiencies using KRED 112 and 124, respectively. Computational modeling of compounds **67** and **68** at the B3LYP/6-31+G(d,p) level of theory suggested significant distortion of the aldehyde carbon toward a tetrahedral geometry, implying that an intramolecular n → π* interaction between the *N*-oxide and aldehyde may result in the lower barriers to rotation observed in these compounds.^69^ The enantioenriched atropisomeric biaryl *N*-oxide products performed well as Lewis base organocatalysts for the asymmetric allylation of aldehydes.

Shortly after the submission of this Account, Lewis and co-workers reported the DKR of 3-aryl-4(3*H*)-quinazolinone derivatives via atroposelective C–H bromination using an engineered flavin-dependent halogenase.^70^ High levels of regioselectivity and stereoselectivity were obtained for a range of compounds, and atropostable starting substrates underwent nondynamic KR. This represents the first example of the use of both a halogenase in atroposelective synthesis and the biocatalytic enantioselective preparation of C–N atropisomers.

Akai and co-workers have reported the two-step deracemization of a range of 2,2′-binaphthol (BINOL) derivatives **71** (Scheme 15).^71^ These 2,2′-BINOL derivatives are conformationally stable but may undergo enantiomerization through the use of a Ru(II) racemization catalyst. The Ru(II) catalyst is proposed to mediate an oxidative SET of the BINOL substrate, generating a free radical on the carbon center at the biaryl axis, which induces conformational lability even at low temperature. Combining this racemization step with a concurrent lipase-catalyzed acylation using isopropenyl acetate **72** gave a range of monoacetates (*R*_a_)-**73** atroposelectively. It is worth noting that although this sequence was characterized as a DKR by the authors, the overall transformation rather falls into the definition of a dynamic kinetic asymmetric transformation (DYKAT), as the starting substrates are conformationally stable and undergo catalyst-mediated rapid interconversion.^18,72^ Monoacetates (*R*_a_)-**73** were subsequently subjected to deacylation by an inorganic base to afford the deracemized diols (*R*_a_)-**71** with high overall yields and enantioselectivities.

**Scheme 16:**
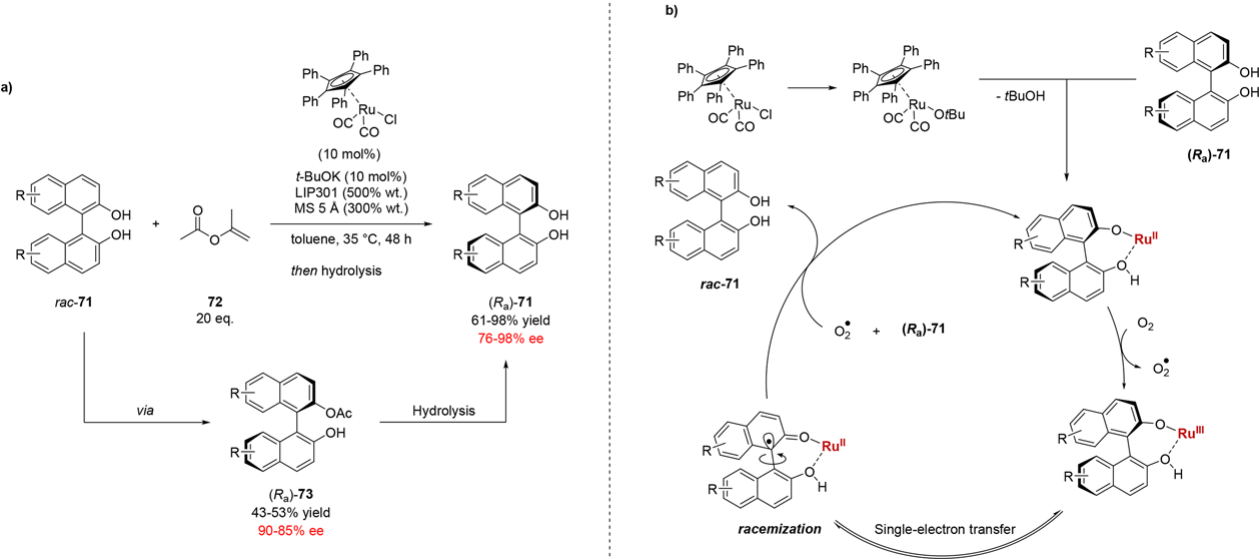
(a) DYKAT of BINOL Derivatives *rac*-**71** by Concurrent Ru(II)-Catalyzed Racemization and Lipase-Mediated Acylation and (b) Proposed Mechanism of the Ru(II)-Catalyzed Racemization

## 6 Conclusions and Prospects

The use of biocatalysts for the enantioselective synthesis of atropisomeric compounds is a promising field of research. Although oxidative couplings are common in biosynthetic pathways, the synthetic potential of these enzymes as biocatalysts is yet to be realized in routine applications. The majority of currently reported strategies rely on the KR of racemic mixtures of conformationally stable compounds or the desymmetrization of prochiral molecules. We have shown that enantioenriched biaryl and nonbiaryl atropisomers, such as arylisoquinoline-*N*-oxides and diaryl ethers, can be accessed by desymmetrization using oxidoreductases. Although ideally suited to the asymmetric synthesis of atropisomers, owing to the the inherent rotational lability of single bonds, biocatalytic dynamic resolution methods remain comparatively underexploited. We have demonstrated that intramolecular noncovalent interactions contributing to low barriers to rotation in heterobiaryls allow biocatalysis to achieve dynamic kinetic resolution, transforming them into products in which these intramolecular interactions are suppressed.

**Figure 4:**
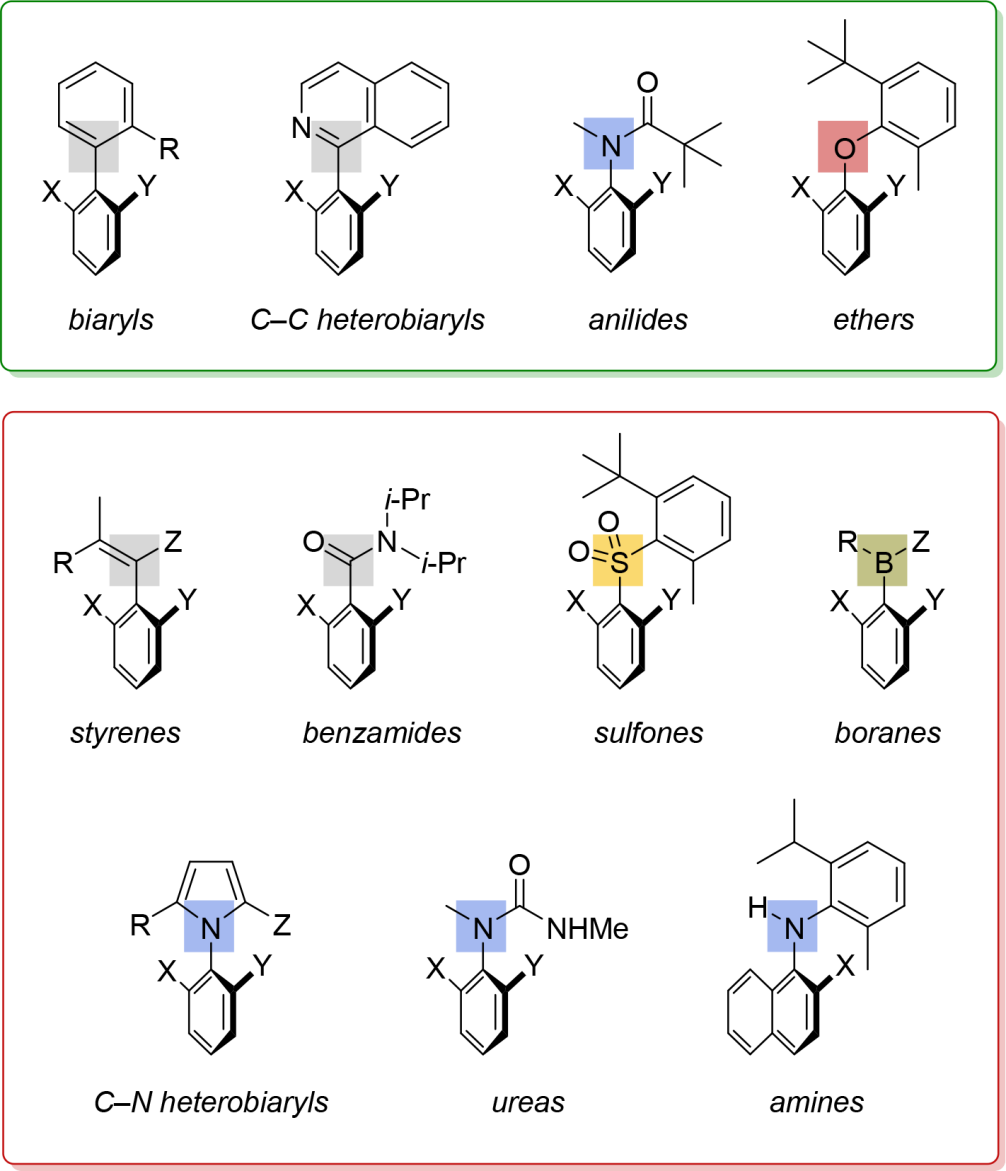
Important classes of atropisomeric compounds that have or have not yet been made enantioselectively by biocatalytic methods (green and red boxes, respectively).

While we and others have reported the biocatalytic enantioselective preparation of axially chiral biaryls, heterobiaryls, and diarylethers, several increasingly important classes of molecules known to exhibit atropisomerism around C–C and C–Het bonds (Het = N, S, B) have not yet been prepared by such means (Figure 4).

It is also interesting that several biocatalytic deracemization strategies, including stereoinversion, linear or cyclic deracemization, and enantioconvergent processes, have been described previously but are yet to be applied to the enantioselective preparation of atropisomers.^73^ Additionally, artificial metalloenzymes (i.e., the artificial combination of a non-native catalytically active metal catalyst within a protein scaffold),^74^ which combine the diverse reactivity of homogeneous metal catalysts with the substrate selectivity and well-defined 3D structures of enzyme active sites, represent a promising approach to the synthesis of enantioenriched atropisomers. For instance, an interesting example of a biocatalytic atroposelective cross-coupling reaction using an artificially evolved “Suzukiase” has been reported,^75^ demonstrating that this field, though still in its infancy, could lead to numerous future applications.

In light of important advances in enzyme discovery and engineering as well as computational design, biocatalysis is increasingly becoming an essential part of the synthetic organic chemist’s toolbox. It is likely that new strategies incorporating biocatalysts will be developed for the stereoselective preparation of atropisomers and more broadly for the control of molecular conformation.
